# Prevalence of cattle flukes infection at Andassa Livestock Research Center in north-west of Ethiopia

**Published:** 2012

**Authors:** Asressa Yeneneh, Hassen Kebede, Tewodros Fentahun, Mersha Chanie

**Affiliations:** 1 *Department of Veterinary Clinical Studies, Faculty of Veterinary Medicine, University of Gondar, Gondar, Ethiopia; *; 2 *Department of Basic Veterinary Science, Faculty of Veterinary Medicine, University of Gondar, Gondar, Ethiopia;*; 3 *Department of Veterinary Paraclinical Studies, Faculty of Veterinary Medicine, University of Gondar, Gondar, Ethiopia.*

**Keywords:** Prevalence, Cattle, Fluke, Ethiopia

## Abstract

A cross sectional study was carried out from October 2010 to March 2011 at Andassa Livestock Research Center, North-West Ethiopia. The objective was to determine the prevalence of cattle flukes infection. Faecal samples were collected from a total of 384 cattle, cross breed (n= 39) and Fogera breed (n=345) of all age groups and sex. Sedimentation technique was employed for the recovery of fluke eggs from freshly collected fecal sample. The results indicated that the overall prevalence of bovine flukes infection was 60.42%. In this study, the highest prevalence was recorded from Paramphistomosis (45.83%) followed by Fasciolosis (23.96%), and Schistosomosis (9.89%). The prevalence of flukes infection was higher in age group 1- 2 years old. There was significant difference in case of Paramphistomosis among age groups. No significant association was found between crossed breeds and sex groups for fluke’s infection. The prevalence of Paramphistomosis was high in cross breed (58.97%) than Fogera breed (44.35%). However, in both cases, there was no significant difference. The result of the present study revealed that the prevalence of major bovine fluke infection in the study area was relatively low and is the definite proof of active infection.

## Introduction

Ethiopia is believed to have the largest livestock population in Africa, yet produces insufficient animal protein and other livestock products to meet the demand of fast growing human population. The contribution of livestock industry to the national economy is considerably less than its tremendous potential. Among many constraints that made the livestock sector marginal is due to prevalent of different diseases, malnutrition, and management constraints. Parasitism represents a major obstacle to the development of the sector.^1^

Flukes of ruminants are flat worm (trematodes) parasites living in liver (*Fasciola, *liver fluke), proventriculus (*paramphistomum,* rumen fluke) or blood (*Schistosoma*, blood fluke).^2^ The life cycles of flukes are always indirect, involving one or two intermediate hosts before invasion of definitive hosts. The snails such as *Lymnaea truncataula* for *Fasciola*; *Planorbis* or *Bulinus* for *paramphistomum* and *Bulinus contortus*, *Physopsis africana*, *Physopsis globsa* and *Physopsis* *nausta *for *Schistosoma *act as intermediate hosts for these flukes.^3^

They are narrowly dependent of their close environment (nature of the soil), and of the climatic conditions for survival and multiplication of the intermediate hosts and also for the survival and evolution of larval stages (miracidium, sporocyst, redia, cercaria, and metacercaria).^2^

Pathogenesis of Fasciolosis varies according to the phase of parasitic development in the liver and species of host involved, essentially the pathogenesis is twofold; the first phase occurs during migration in the liver parenchyma and is associated with liver damage and hemorrhage. Early infection, during fluke migration, there is hyperproteinemia, hyperglobulinemia, and hypo-albuminemia. The hypoalbuminemia is associated with plasma volume expansion caused by liver damage and reduced albumin synthesis. The second phase occurs when the parasite is in the bile ducts, and results from the hematophagic activity of the adult flukes and from the damage to the mucosa, by their cuticular spines.^4^

The adult Paramphistomum in proventriculus is essentially non-pathogenic even though large numbers may present. At most there may be a localized loss of rumen papillae. The immature helminths attach to the duodenal mucosa by means of posterior suckers and causes severe enteritis, possibly necrosis and hemorrhage. In heavy infestation a frank hemorrhage, duodenitis, hypoproteinemia and edema may be produced with immature flukes deeply embedded in the mucosa. Severely Affected animals exhibit anorexia, polydipsia, unthriftiness and severe diarrhea.^5^

*Schistosomes* are found in the portal and mesenteric blood vessels, and the principal clinical signs are associated with passage of the spined eggs through the tissue of the gut lumen. The young parasites cause some damage during migration, but most of the lesions are due to the irritation produced by the eggs of parasites in the intestine and other organs, and blood sucking habit of the helminthes worm. The helminthes worm may also enter the vesical veins and they may cause hematuria.^6^

Even though there are different research works conducted in relation to bovine Fasciolosis and Schistosomiasis in different parts of Ethiopia,^7^^-^^13^ no research works touched bovine Paramphistomiasis; despite its significance in veterinary practice. This study aims to fill such gap and it has been carried out in cattle at Andassa Livestock Research Center. Therefore, the objectives of current study were to identify and explore the status of major flukes diseases prevalent in the study area by classical coprological examination and to asses some of the epidemiological risk factors that might contribute for fluke infections.

## Materials and Methods

**Study** **area**. This study was conducted at Andassa livestock Research Center (ALRC) (11^° ^29' N and 37^° ^29' E) with 1,730 meters above sea level starting from October 2010 to March 2011. It is located in Amhara regional state, western Gojjam zone, Bahir Dar Zuria woreda. It is 587 km away from Addis Ababa in North West direction and 17 km away from Bahir Dar town in south direction. It receives average annual rainfall of 1150mm with the mean annual temperature varies from 8.8 ^°^C to 29.5 ^°^C. Moreover, the center compound covers 360 hectares, out of which 310 is covered by pasture land (grazing land and hay preparation) and 50 hectares is covered with bush. The soil is dominantly characterized by dark clay soil, which is seasonally water logged in the rainy season and cracked when dry. The topography of the area varies from a river valley plain to a gentle slope of grass land. The dominant grass vegetation of the area includes Cynodon, Hyperhenia, Andropogon, Paspalum, Cetaria, Elusin, Eragrostis, Sporobulus and Trifolium species.^14^

**Study** **population. **The examined animals were pure Fogera cattle and its crosses with Holstein Friesian reared at Andassa livestock research center. The center has a total of 500 cattle (both pure Fogera and its crosses with Holstein Friesian). The Fogera breed of cattle is considered as a definite breed, having its own characteristics. The breed originates from the area around Lake Tana in south Gondar administrative zones. 

**Herd** **management. **The quantity of milk that can be produced by cow depends primarily on three factors -breed, management and nutrition.^10^ Sometimes concentrate feeds are supplemented for milking cows, bulls and sick animals. The animals were watered from Andassa River and spring water during wet season for young and sick animals which stayed at barn. One of the greatest ravages of the profits of dairy farms is disease.

Appropriate herd health management practices play great roles in inducing the individual animals as well as herds to disease^14^. Since animals are watered from Andassa River, gastrointestinal parasites are common. As a result of seasonal deworming was practiced at the beginning and ending of rainy season using appropriate anthelmintics.

In the breeding program, both natural mating with Fogera bulls and artificial insemination with Holstein Friesian semen were used. Cattle were managed in a loose housing system. Calves had free access to suckle their dams for the first four days to ensure that they consume enough colostrum. They were then separated from their dams and allowed to partially suckle (two teats) at milking times until weaning. Calves stayed around the barn until three months of age and allowed for grazing thereafter. While they were at barn they were provided with hay and water.

**Study design and sample size** **. **A cross –sectional study on fluke infection was carried out from October 2010 to March 2011 in Fogera cattle and its cross lines with Holstein Friesian at Andassa Livestock Research Center. The study site was selected purposively while study animals were sampled by using simple random sampling technique and a total of 384 cattle were sampled. The sample size for this study purpose was determined according to Thrusfield.^15^ Since no previous study conducted on cattle fluke infection in the study area, 50% expected prevalence was considered during sample size determination. The other determinants considered in sample size determination are 95% confidence internal and 5% desired absolute precision. 

**Data** **Recording.** While collecting fecal samples from study animals, all data was recorded with pre-designed format and entered to computer using Microsoft excel spreadsheet. The individual animal details such as animal ID, sex, age and breed were also registered together. 

**Fecal examination**. While the initial recording of the animal detail is taken, fecal samples (approximately 10 gram) were collected directly from the rectum of the animal. The fecal sample was then put into 10% formalin filled universal sampling bottle. After labeling with specific identification number, each sample was transported to Bahir Dar Animal Health Investigation and Diagnostic laboratory, Parasitology department for coprological examination. Sedimentation technique was employed to assess the presence of trematode eggs through repeated dilution of the fecal suspension and sedimentation of the eggs, which are heavier than most of the fecal particles.^16^ All collected fecal samples were examined at Bahir Dar Animal Health Investigation and Diagnostic laboratory, Parasitology department.

**Statistical Analysis** ***. ***The data were analyzed using SPSS software version 17 (SPSS Inc., Chicago, Illinois, USA). The association between prevalence and examined animals’ data (age, sex, and breed) were evaluated using Chi-square (^2^) test.

## Results

**Overall prevalence**. A total of 384 fecal samples were collected and examined. Out of these 232 cattle were found to be positive for fluke eggs with overall prevalence of 60.42%. Of those, 23.96%, 45.83%, and 9.89% was found to be infected with *Fasciola, Paramphistmum*, and *Schistosoma*, respectively. Higher percentage was recorded for *Paramphistomum* followed by *Fasciola* and *Schistosoma *(Table 1).

**Age specific prevalence** **.** The prevalence of bovine Fascioliasis, Paramphistomiasis and Schistosomiasis was higher in age group between one and two years (Fascioliasis, 24.24%; Paramphistomiasis, 53.03% and Schistosomiasis, 10.61%) than that of age groups bellow one year (Fascioliasis, 15.79%; Paramphistomiasis, 22.37% and Schistosomiasis, 10.53%) and above two years (Fascioliasis, 21.90%; Paramphistomiasis, 51.24% and Schistosomiasis, 9.50%). There was high significance difference of Paramphistomiasis between age groups (χ^2 ^= 21.081, p = 0.000). However, no any significance difference in Fasciolosis (χ^2^=1.773, p=0.412) and Schistosomiasis (χ^2^=0.113, p=0.945) (Table 1).

**Sex specific prevalence** **.** The prevalence of bovine Fasciolosis and Schistosomosis was higher in female (Schistosomosis, 11.22%; Fasciolosis, 21.45%) than that of male (Schistosomosis, 4.94%; Fasciolosis, 19.75%). On the other hand bovine Paramphistomiasis prevalence was higher in male (48.15%) than female (45.21%). In both cases there was no significance difference between two sex groups (Fasciolosis: χ^2^=0.111, p=0.739; Paramphistomosis: χ^2^=0.222, p=0.638 and Schistosomosis: χ^2^=2.830, p=0.093) (Table 1).

**Breed specific prevalence** **. **The prevalence of Fasciolosis and Schistosomiasis were found also higher in Fogera cattle (Fasciolosis, 21.16% and Schistosomiasis, 10.43%) than that of cross breed cattle (Fogera × *Holstein-Friesian*) (Fasciolosis, 20.51% and Schistosomiasis, 5.13%) whereas the prevalence of Paramphistomiasis was higher in cross breed (58.97%) than that of Fogera cattle(44. 35%).There was no significant differences between bovine fluke infections (Fasciolosis: χ^2^=0.009, p=0.925; Paramphistomiasis: χ^2^=3.019, p=0.082 and Schistosomiasis: χ^2^=1.107, p=0.293) and breeds (Table 1).

**Table 1:** Prevalence (%) of flukes infection with respect to age, sex, and breed of animals

**Variables**	***Fasciola***			***Paramphistomum***			***Schistosoma***		
	**Prevalence**	**χ** ^2^	***P*** **-value**	**Prevalence**	χ^2^	***P*** **-value**	**Prevalence**	**χ** ^2^	***P*** **-value**
**Age (year)**									
≤ 1 (n=76)	15.79			22.37			10.53		
1< x ≤2 (n=66)	24.24	1.773	0.412	53.03	21.08	0.000	10.61	0.113	0.945
> 2 (n=242)	21.90			51.24			9.50		
**Sex**									
Male (n=81)	19.75	0.111	0.739	48.15	0.222	0.638	4.94	2.830	0.093
Female (n=303)	21.45			45.21%			11.22		
**Breed**									
Fogera (n=345)	21.16	0.009	0.925	44.35	3.019	0.082	10.43	1.107	0.293
Cross (n=39)	20.51			58.97			5.13		

n=number of animals examined, χ^2^ = Chi square

## Discussion

The present study indicated that the overall prevalence of cattle fluke infection at Andassa Livestock Research Center was high. The highest prevalence was recorded from Paramphistomosis followed by Fasciolosis and Schistosomosis. The differences among the prevalence of flukes’ infection might be attributed due to the biology of the parasite and egg detection techniques of flukes. The higher prevalence of *Paramphistomum* infection may account partly by no effective treatment non-pathogenic helminth, and numerous intermediate hosts. Moreover, Adult *Paramphistome* is very prolific and many eggs are expelled.^2^ The prevalence of Paramphistome was higher than prevalence recorded from dairy cattle of Central France (15.00%).^17^ Prevalence of Fasciolosis and Schistosomiasis infections was highly lower than those from other parts of the country (61.00% in Ethiopia,^10^ 52.00% from Gondar clinic,^9^ 47.10%,^13^ 36.72 %,^18^ and 33.8%^10^ in Bahir Dar, and 22.06%^8^ from in and around Bahir Dar on *Schistosomia*). This discrepancy might be attributed due to differences in ecological, climate conditions, sample size, and management systems. 

In this study, fluke infections were highly prevalent in age groups 1- 2 years old. There was significant difference between age groups. This finding is in agreement with earlier studies^11^^, ^^18^^-^^20^ who reported that fluke infection was low in calf group (≤1 years age). This is attributed to the fact that calves are not often driven with older age groups to grazing and watering points. On the other hand, the current finding recorded relatively low prevalence of fluke infection of ages (> 2 years) than that of age group of cattle between 1 and 2 years. This finding is unlikely with the works carried out in different parts of the country.^11^^,^^18^^- ^^20^ This is attributed to the fact that older animals can develop resistance to re infection.^3^

The prevalence of fluke infections was not significantly associate with animal sex. This finding is in accordance with other researches.^11^^,^^18^^,^^19^ This may be due to cattle in the research center are driven to pasture land and watering points regardless of sex even if, animals are belonging to separated herds.

The prevalence of Fasciolosis and Schistosomosis were relatively higher in Fogera cattle than cross breed cattle. This finding disagrees with the result recorded by Hailu who noted that Fogera breed is known for its tolerance to parasitic diseases.^10^ There was no significance difference between bovine fluke infections and breeds. These findings also disagree with the result described by Fikirtemariam.^18^ The reason may be associated with imbalanced sampling ratio; small sample size of cross breed (n= 39) and relatively large sample size of Fogera breed (n=345) and better management (improved feed supply) to cross breed in the research center.

The present study demonstrated that the overall prevalence of major bovine fluke infections at Andassa Livestock Research Center (ALRC) is remarkably high. Even though, the prevalence of *Fasciola*, *Paramphistomum* and S*chistosoma* is relatively low compared to the previous studies under taken in different parts of the country, positive result of fluke infection is the definite proof of an active infection and a focus of infection for the herd with eggs being released onto the pastures and infecting the snails. The major feed resources at ALRC are almost natural pasture in the form of grazing land which is seasonally water logged and the research center lack clean piped water to animals (animals are watered at Andassa River) increasing the chance of exposure to fluke infection. Moreover, epidemiologically the area is favorable for the development and multiplication of intermediate hosts. Accordingly, strategic application of fluckicide and provision of worm safe pasture and water provide better considerable success in the prevention/control of fluke infection in the study area. 

Due to limited accuracy of coprological examination, it should be supported by other diagnostic techniques like post-mortum and immune diagnosis so as to provide a clear picture on the prevalence of bovine fluke infection in the study area. The role of different epidemiological factors such as, age, sex, breed, season and the type of intermediate hosts involved in the prevalence of fluke infections should clearly be established in order to understand their effect in the control of fluke disease in the future.
